# TRainee Attributable & Automatable Care Evaluations in Real-time (TRACERs): A Scalable Approach for Linking Education to Patient Care

**DOI:** 10.5334/pme.1013

**Published:** 2023-05-17

**Authors:** Jesse Burk-Rafel, Stefanie S. Sebok-Syer, Sally A. Santen, Joshua Jiang, Holly A. Caretta-Weyer, Eduardo Iturrate, Matthew Kelleher, Eric J. Warm, Daniel J. Schumacher, Benjamin Kinnear

**Affiliations:** 1 Division of Hospital Medicine, NYU Langone Health, and assistant director of Precision Medical Education, Institute for Innovations in Medical Education, NYU Grossman School of Medicine, New York, USA; 2 Department of Emergency Medicine, Stanford University School of Medicine, Stanford, California, USA; 3 University of Cincinnati College of Medicine, Cincinnati, Ohio, USA; 4 University of California Los Angeles, Los Angeles, California. At the time of this work he was a medical student, NYU Grossman School of Medicine, New York, USA; 5 Data Core, NYU Langone Health, New York, USA; 6 Internal Medicine and Pediatrics, Department of Pediatrics, Cincinnati Children’s Hospital Medical Center, University of Cincinnati College of Medicine, Cincinnati, Ohio, USA; 7 Department of Pediatrics, director of Education Research Unit, Cincinnati Children’s Hospital Medical Center/ University of Cincinnati College of Medicine, Cincinnati, Ohio, USA

## Abstract

Competency-based medical education (CBME) is an outcomes-based approach to education and assessment that focuses on what competencies trainees need to learn in order to provide effective patient care. Despite this goal of providing quality patient care, trainees rarely receive measures of their clinical performance. This is problematic because defining a trainee’s learning progression requires measuring their clinical performance. Traditional clinical performance measures (CPMs) are often met with skepticism from trainees given their poor individual-level attribution. Resident-sensitive quality measures (RSQMs) are attributable to individuals, but lack the expeditiousness needed to deliver timely feedback and can be difficult to automate at scale across programs. In this eye opener, the authors present a conceptual framework for a new type of measure – TRainee Attributable & Automatable Care Evaluations in Real-time (TRACERs) – attuned to both automation and trainee attribution as the next evolutionary step in linking education to patient care. TRACERs have five defining characteristics: *meaningful* (for patient care and trainees), *attributable* (sufficiently to the trainee of interest), *automatable* (minimal human input once fully implemented), *scalable* (across electronic health records [EHRs] and training environments), and *real-time* (amenable to formative educational feedback loops). Ideally, TRACERs optimize all five characteristics to the greatest degree possible. TRACERs are uniquely focused on measures of clinical performance that are captured in the EHR, whether routinely collected or generated using sophisticated analytics, and are intended to complement (not replace) other sources of assessment data. TRACERs have the potential to contribute to a national system of high-density, trainee-attributable, patient-centered outcome measures.

## Introduction

Competency-based medical education (CBME) is an outcomes-based approach to clinical training [1,2,3], yet empirical evidence demonstrating the impact of this approach on patient outcomes is lacking [4,5]. Trainees are often sheltered from (or generally unaware of) measures that reflect their clinical performance, making the quality of the care they provide obscure to themselves and their training programs. We use the broader term ‘trainee,’ which includes health professions students, residents, and fellows, to emphasize that this gap exists across the training continuum. Institutional and governmental stakeholders have encouraged linking medical education with patient outcomes [6,7,8,9]. While some studies have successfully linked education to patient outcomes after training [10,11,12,13], identifying and implementing such linkages *during training* remains underexplored [14]. This shortcoming limits our ability to provide tailored educational experiences, timely feedback, targeted coaching, and defensible entrustment decisions [15,16]. Furthermore, trainees are demanding greater precision in the data they receive and expect that any feedback provided about their clinical performance will be supported with data [17]. The existing gap between educational and patient outcomes is increasingly regarded as a significant limitation of CBME [4,5].

Many educational assessments (e.g., standardized testing and end-of-rotation evaluations) lack validity evidence that demonstrates these measures reflect or predict the quality of care delivered by trainees [13,18,19,20]. In contrast, clinical care measures are focused on the patient and typically encompass multiple factors attributable to the individual trainee, team, patient, and health system, but without clear distinctions [21]. In 2010, Kalet and colleagues introduced the concept of an educationally sensitive patient outcome (ESPO) in an attempt to ‘link educational interventions to patient health outcomes’ [22]. Proposed ESPOs include patient activation, clinical microsystem activation, and health literacy—all of which theoretically are affected by changes and differences within a trainee’s education [22,23]. A major limitation of ESPOs, however, is that the implementation requires a labor-intensive data-gathering process. For instance, patient activation requires a motivational interviewing intervention and measurement using a Patient Activation Measure [24,25].

Clinical performance measures (CPMs) and electronic clinical quality measures (eCQMs) reflect important patient process or outcome measures at scale [26]; however, most CPMs/eCQMs are limited in their attribution to individual providers, not to mention trainees who are known to be interdependent with their supervisors [27]. For example, the eCQM assessing the percentage of patients with poor outpatient glycemic control (hemoglobin A1c >9%) is a provider-attributed outcome that cannot be attributed to a single individual. Rather, this measure reflects the contributions of several team members, systems, and patients themselves. Attention to individual attribution is a necessary evolutionary step in advancing the science around the use of clinical measures in education.

The introduction of resident-sensitive quality measures (RSQMs) aimed to develop measures that are clinically important and substantially attributable to individual trainees. RSQMs leverage information from the electronic health record (EHR) and prioritize measures attributable to individual trainees, often using process measures (e.g., orders, clinical documentation) that are more proximal to an individual’s actions and behaviors [28]. For example, RSQMs proposed by Kinnear et al. for the inpatient internal medicine setting include: documentation of diabetes classification, adjusted insulin dose at least every 24 hours with consistent hyperglycemia, and documented changes to home insulin regimen in the discharge summary [29]. Moreover, RSQMs function as both program- and system-level measures when calculated in aggregate across trainee cohorts. However, RSQMs were not designed with scalability or automation in mind, thus limiting their implementation across clinical settings.

Therefore, the aim of this eye opener is to offer a new conceptual framework for assessing the clinical performance of trainees in a way that is scalable and can inform team- and system-level outcomes.

## The Eye Opener: A TRACER Conceptual Framework

To fulfill the promise of CBME, high-density, high-quality data about trainees’ clinical performance are needed across the educational continuum. We assert that designing measures of trainees’ clinical care that combine automation, scalability, trainee attribution, and timeliness are necessary to accomplish CBME. To that end, we offer TRainee Attributable & Automatable Care Evaluations in Real-time (TRACERs) as a conceptual framework for advancing the science around clinical measures for trainees. TRACERs are clinical performance measures that prioritize automation and scalability while focusing on trainee attribution and near real-time feedback loops. TRACERs are an evolution of previous attempts to measure trainees’ clinical outcomes that combines the philosophy of ESPOs with the principles of both CPMs and RSQMs to assess the care provided by trainees across the medical education continuum in a scalable, timely, and trainee-attributable fashion.

**Figure 1:**
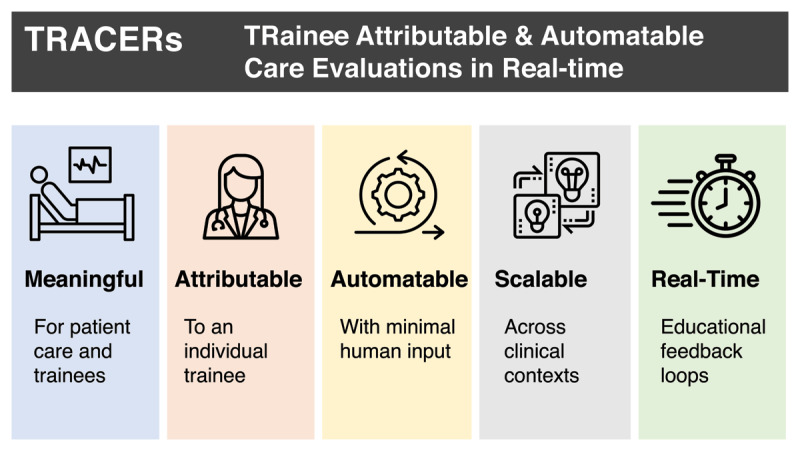
TRainee Attributable & Automatable Care Evaluations in Real-time (TRACER) conceptual framework.

TRACERs are developed by identifying clinical scenarios where trainees provide care, extracting information from the EHR, and calculating completion rates for different care measures. TRACERs can analyze processes (e.g., guideline-directed ordering of diabetes medications for inpatients) and outcomes (e.g., hyperglycemic events linked to non-guideline-directed ordering of diabetes medications for inpatients). They are guided by five distinct concepts each with defining characteristics (Figure 1).

### Meaningful

TRACERs should reflect a *meaningful* component of clinical care, whether at the patient or system level. Process measures should have a clear rationale for ‘how’ and ‘why’ they impact patient outcomes. For example, evidence exists that the use of basal/bolus insulin strategies (rather than simply sliding scale insulin) for adult inpatients with insulin-dependent diabetes mellitus leads to better glycemic control [30]. A process-based TRACER assessing insulin ordering behaviors among trainees admitting patients with diabetes would have a strong evidence-based justification. Likewise, outcome measures should be recognized as important markers of patient care. For example, glycemic control has been shown to impact patient morbidity and mortality [30]. But, meaningfulness needs to go beyond the clinical outcomes. TRACERs must also be educationally meaningful for trainees, reflect credible measures [31] of clinical performance for which trainees perceive a substantial degree of responsibility (see attribution discussed below) [32], and be educationally sensitive (i.e., the measure could be useful for informing behavior change) [22]. Ideally, TRACERs should consist of processes or outcomes that occur frequently enough to provide ample opportunities for feedback and sufficient power for any data analysis to quantify growth curves over time.

### Attributable

TRACERs need to measure actions that are substantially *attributable* to a single trainee. To identify TRACERs with substantial attribution, appropriate clinical scenarios must be selected for which trainee-attributable data exist. A given TRACER’s degree of attribution to an individual may vary by that individual’s role on the care team [33], their stage of training, and clinical context (e.g., time of day, day of the week). For instance, TRACERs that involve medication orders might be highly attributable for an intern, slightly less attributable for a medical student sub-intern (depending on how much autonomy they receive and how many orders they enter for others to co-sign), and not very attributable to a clerkship student. In contrast, TRACERs developed related to admission notes – such as documentation of an explicit prioritized differential diagnosis [34,35] – may be equally amenable for students, sub-interns, and residents, who all document admission notes. Therefore, while TRACERs can be used across the education continuum, identifying moments where attribution is more likely to exist for a given trainee is imperative to maximizing specificity. Metrics which incorrectly identify patients and scenarios for inclusion in their measurement (i.e., low specificity) are likely to be discounted by trainees when used for formative purposes [36,37], and could potentially damage the educational alliance by eroding a trainee’s trust in the assessment system.

### Automatable

TRACERs – once developed, tested, and implemented by humans – should be generated *automatically* based on readily available machine-readable EHR data. Automation is critical for collecting high-density trainee performance data, and necessary, but not sufficient, to enable real-time feedback loops (discussed further below). To achieve automation, clinical informatics techniques such as database queries and advanced analytics can identify appropriate clinical scenarios according to pre-defined parameters and collate relevant trainee-attributable data. Once implemented, TRACERs should minimize reliance on human oversight to decide whether the metric has been completed. By having TRACERs rely on computer algorithms to determine whether a given action has been taken by the trainee, we can assess trainees with higher accuracy and precision and alleviate the assessment burden among clinical supervisors [38]. Reliance on machine-readable EHR data – which can include both structured and unstructured data – limits which competencies TRACERs can assess. For example, TRACERs are well positioned to measure the clinical actions of trainees, which are captured in the EHR, but are less likely to assess their physical examination skills. TRACERs are valuable for identifying moments of independence and have the potential to complement other forms of assessment data. Work-based assessment efforts can, and should, continue as a key tool for programmatic assessment, particularly with physical exam skills or other humanistic aspects of patient care.

### Scalable

TRACERs present an opportunity for sharing assessment approaches across institutions rather than being siloed within single programs. Particularly as EHRs are becoming ubiquitous in medicine, *scalability* represents both a challenge and an opportunity for education. Ideally, TRACERs should be replicable across EHRs and clinical environments. Pragmatically, achieving such scalability is difficult, as EHR vendors, data warehouses, and database query languages often differ across (and sometimes within) institutions. Also, clinical workflows can change frequently, sometimes even across shifts within a single setting. Efforts towards scalability may take the form of open-source database queries applied to a well-defined context, which can be retooled to execute in a wide range of possible training settings. EHR platforms are increasingly building cloud-based solutions to facilitate the interoperable sharing of complex models [39,40,41]. TRACERs leverage these innovative approaches to enhance scalability. Fundamentally, scalability depends on fastidious validation efforts across settings and EHRs to ensure that TRACERs, though customizable to a degree, remain accurate in their local context. Emerging data standards and guidelines [42] are critical for ensuring robust validation across contexts.

### Real-time

TRACERs offer a *real-time* (or near real-time) mechanism for informed self-assessment; they also allow educators to engage in feedback conversations that can support improvements in trainees’ educational trajectories. Trainees value timely feedback because it allows them to reflect on their clinical actions and decisions [43], including encounter-, team-, and system-level factors which are critical to contextualize one’s performance. TRACERs’ timeliness affords such contextualization, which maintains credibility in the eyes of trainees when scaling across diverse clinical environments where local factors may impact accuracy. However, automation is necessary but not sufficient for meaningful real-time feedback loops. Measures must also occur throughout training with sufficient frequency to be estimated reliably in real-time at the individual trainee level and to facilitate iterative behavior change by trainees [44]. Notably, focusing on measures amenable to real-time feedback may limit TRACERs to largely proximal process and outcome measures. More temporally distal measures may nonetheless be relevant to trainees and worth developing, though would by definition lack real-time feedback loops.

## Discussion

Assessment requires making design choices and balancing trade-offs. TRACERs are no different in this regard. While the ideal TRACER would fully meet each of the aforementioned criteria, in reality each characteristic may be weighted and prioritized more than another depending on context. For example, an artificial intelligence-based automated assessment of clinical reasoning documentation quality in native clinical notes [35] is meaningful, automated, highly attributable, and real-time but may lack scalability to other contexts that use different note structures. Having a combination of TRACERs that are designed to optimize different aspects affords us a more precise way of accounting for individual-, team-, and system-level aspects.

**Figure 2:**
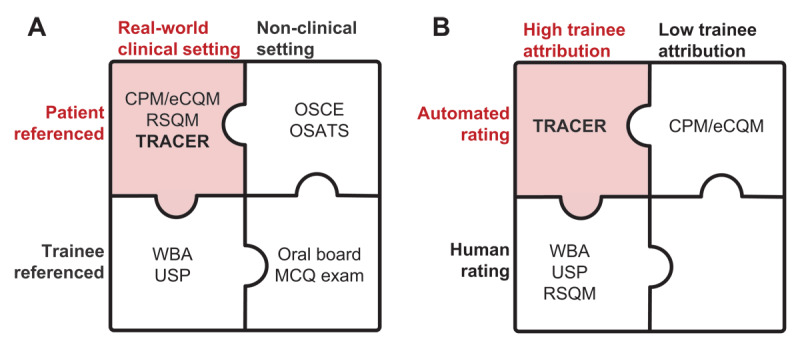
**Complementary approaches to assessing the clinical performance of individual physicians in training in a program of assessment. (A)** Clinical performance measures (CPMs), electronic clinical quality measures (eCQM), resident-sensitive quality measures (RSQMs), and TRainee Attributable & Automatable Care Evaluations in Real-time (TRACERs) are distinguished from other assessments in their use of clinical care data to assess physician performance in the real-world clinical setting with a patient frame of reference. In contrast, work-based assessments (WBAs), unannounced standardized patient (USP) programs, observed structured clinical examinations (OSCEs) and group OSCEs, observed structured assessments of technical skill (OSATS), oral board exams, and multiple-choice question (MCQ) exams either take a trainee frame of reference or occur in a simulated/non-clinical setting. **(B)** Among assessments in the real-world clinical setting, TRACERs are distinguished by their combined focus on attribution to individual trainees and amenability to automation, with limited human input when fully implemented.

TRACERs can complement other clinically focused assessments of individuals to provide a more accurate representation of trainees’ performance in clinical environments. TRACERs share with CPMs and RSQMs the patient as a frame of reference in the real-world clinical environment (Figure 2A). Like CPMs/eCQMs, TRACERs account for automation and scalability during the development process, aiming to maintain natural clinical workflows, draw upon existing EHR-derived clinical data, and minimize manual data retrieval. Placing these requirements on TRACERs maximizes their scalability across the spectrum of training sites, potentially enabling the medical education community to evaluate individual- and program-level outcomes at a national level. TRACERs draw upon the attribution lessons of RSQMs, yet broaden the scope to include all trainees who interact with the EHR. While RSQMs could be defined for these groups and appropriately named to reflect this, TRACERs explicitly take this additional step. Medical education does not start or end with residency; tracking longitudinal outcome measures establishes a foundation upon which educators may analyze curricular efficacy, and TRACERs could be one element of a trainee’s portfolio of outcomes that transcend training program boundaries [45]. Among assessments in the real-world clinical setting, TRACERs are unique in their combined attention to automation and attribution (Figure 2). Finally, TRACERs are not limited to traditional domains of quality (e.g., health outcomes and clinical processes), but are rather envisioned as process or outcome measures related to clinical care more broadly, including patient safety, resource utilization, and care coordination.

To illustrate the distinction, Table 1 compares a diabetes-focused traditional CPM [46] and a sample TRACER along the core characteristics of TRACERs. Two educationally important categories – meaningfulness for trainees and attribution – are not met by the CPM. While average hemoglobin A1c measures are important, the CPM lacks the specificity to the trainee that a TRACER achieves, as it does not reveal specific improvements that a trainee could undertake and cannot serve as a real-time feedback tool. While designed for an internal medicine context, the same TRACER might also apply to management of patients with diabetes on surgical services who are receiving post-operative care. Likewise, among post-laparoscopic cholecystectomy patients cared for by general surgical residents, one might formulate a TRACER targeting guideline-based judicious opioid prescribing on discharge – a patient care behavior that is meaningful [47], attributable, automatable, scalable, and amenable to near real-time feedback. In thinking about what these TRACERs look like within individual specialties, it is important to be mindful of what trainees are doing in the clinical workplace and how those actions and decisions are currently captured. For example, in some institutions opioid prescribing on discharge may be highly protocolized while in others it is at the discretion of the prescriber. Ultimately, TRACERs should reflect care that occurs frequently, could be improved with feedback, and would align with aspects of patient care that are valuable to focus on throughout one’s medical training and practice.

**Table 1:** Comparing a diabetes-focused Traditional Clinical Performance Measure (CPM) vs. a TRainee Attributable & Automatable Care-Evaluation in Real-time (TRACER). Shaded areas indicate characteristic not met.


	CPM	TRACER

	PERCENTAGE OF OUTPATIENTS WITH HEMOGLOBIN A1C (HBA1C) >9.0%	ORDER LONG-ACTING INSULIN FOR INPATIENTS WITH TYPE 2 DM AT RISK FOR HYPERGLYCEMIA

**Meaningful(for patient care)**	Important outcome for patients	Guideline-directed behavior known to improve glycemic control

**Meaningful(for trainees)**	Does not provide trainees with feedback on which aspects of their practices affect HbA1c	Indicates trainee understanding of diabetes and insulin types

**Attributable**	HbA1c levels are product of individual, patient, and system-level factors and may not be predominantly attributable to a single trainee	Reasonably attributable to trainee placing medication orders

**Automatable**	HbA1c automatically calculated from electronic health record panel data	Medication orders recorded by and retrievable from electronic health record

**Scalable**	Employable across clinical settings	Applicable in diverse institutional settings

**Real-time**	Relatively sparse measure that requires a minimum of 3 months to accrue updates and may not drive just-in-time feedback	Amenable to near real-time feedback


## Considerations for Implementing TRACERs

TRACER development and implementation is a multi-step process that requires input from various stakeholders. We recommend first engaging local program leaders (for any specialty) and assessment experts to explore interest in TRACERs. Once support is garnered, collaborating with local clinical and educational experts as well as trainees to explore clinical measures that are both meaningful and reasonably attributable to learners is imperative to ensure buy-in. These measures can be identified through a structured consensus process [29] or through iterative discussion. Next, coordinating with individuals who have operational EHR expertise (e.g., clinical informaticists or data scientists) can help identify which measures can be practically queried from the EHR in an automated fashion. Results can be validated through manual chart review to ensure accurate query performance and proper trainee attribution prior to full implementation. These queries (once validated) can be shared and adapted across clinical environments and institutions. This process is applicable across specialties and clinical environments to create TRACERs that are fit for purpose.

TRACERs ought to be utilized primarily for formative rather than summative purposes. TRACERs could be mapped onto the underlying knowledge, skills, and attitudes required to complete them, and also allow for deliberate practice and improvement. A key element of TRACER validation is assessing how their use in formative feedback loops and coaching drives improvements in patient care – whether assessed through the TRACER itself or other related outcome measures. Moreover, some real-time measures could be assessed for impact during the index patient encounter (e.g., if a trainee sees a TRACER alert for a gap in guideline-directed care, they may be able to address that gap during the same patient encounter if they trust the accuracy of TRACER feedback). However, unlike system-wide best practice advisories (BPA) and clinical decision support (CDS) tools built into the EHR, formative TRACERs are not envisioned as interruptions in patient care.

As TRACERs mature and more validity evidence is collected, their role in summative assessment and use by other stakeholders (such as clinical governance committees, quality improvement groups, institutional leaders, and patient groups) may expand. However, this shift from formative to summative must be cautious and intentional. Over-incentivizing performance on any one metric can lead to inappropriate patient care as providers begin to ‘treat the measure, not the patient’ [48,49]. Such ‘measurement fixation’ can lead providers to lose sight of the larger goal of improving patient care, especially when targeting process measures [50,51]. Implementing TRACERs for formative coaching with appropriate balancing measures [52] may mitigate any unintended consequences of capturing clinical performance. For example, if measuring a trainee’s rate of ordering long-acting insulin for patients with diabetes mellitus, measuring hypoglycemic events that might result from over-ordering of insulin would be an important balancing measure.

As previously discussed, the initiating clinical scenario for a TRACER should occur with enough frequency for each trainee so that performance can be reliably assessed, with future opportunities for improvement identified longitudinally. TRACERs capturing uniformly high performance across a cohort may have utility in providing positive feedback to trainees and engendering agency by highlighting their important role in a team. However, most TRACERs should capture variability in performance across trainees or cohorts – identifying opportunities for improvement among some individuals or areas of achieved competence for others. Bundling several TRACERs together within a single patient care condition or domain may lead to more robust point estimates of each individual’s performance and highlight team- and training cohort-level variation, as has been shown for RSQMs [53]. TRACERs that span typically discontinuous stages of training – such as measuring the same domain during sub-internship and internship – may offer unique insights and create a developmental trajectory that can more precisely identify growth and performance. However, as noted above, attribution may vary by role within a team as well as the conditions that facilitate or impede performance [54].

In feedback sessions with trainees, educators should help learners develop specific improvement activities and be open to discussing TRACER limitations, such as blurring of attribution and contribution in certain clinical scenarios. Faculty training regarding how to integrate TRACERs into coaching discussions that are useful for trainees will be crucial for successful implementation. Existing research examining emergency medicine residents’ feedback conversations when receiving an EHR-based report card in a formative setting showed that trainees can use such measures as learning opportunities, recognizing their limitations, yet contextualizing them with respect to concepts such as attribution/contribution and independence/interdependence [43]. Both trainees and faculty perceive measures derived from the EHR as ‘objective,’ ‘credible,’ ‘valid,’ and ‘complementary’ to existing assessment data. Leveraging existing perceptions of EHR data and providing trainees with opportunities to explore the underlying cases comprising their TRACERs can lead to a paradigm shift in the way we assess individual trainees working in collaborative, team-based contexts.

While TRACERs are designed to be scalable across clinical environments, validity evidence needs to be gathered to support implementation across settings. With current argument-based conceptualizations of validity, evidence is context-dependent [55], and therefore implementation of any assessment approach in a new context will require some validation work. Important validity evidence for TRACERs will include the accuracy of EHR queries, resident attribution (given variability in clinical workflow in different settings), and trustworthiness of the data to support trainees’ learning and growth. Some educators have cautioned against over-reliance on ‘outcomes research’ in medical education and the difficulty in claiming causality in such work [56,57]. However, we would argue that approaches for creating high-density, patient-focused formative assessments of trainees (such as TRACERs) are sorely lacking from most programs of assessment. Developing these measures would complement, not replace, other measures. Likewise, the effort and resources to collect validity evidence for TRACERs are necessary to fulfill their potential to link education with patient care.

TRACERs represent an innovative assessment approach within a broader program of assessment, but they are not the panacea. Given their dependence on EHR data, TRACERs are positioned to assess certain competencies (e.g., patient care or systems-based practice) better than others (e.g., physical examination or verbal communication). TRACERs may be harder to implement than broad, program-level CPMs. Most TRACERs will be particular to a given specialty and setting (i.e., inpatient, outpatient, emergency department, operating room). We suspect that settings with smaller teams (e.g., outpatient clinics) may have clearer trainee-level attribution, potentially making TRACERs more feasible to implement in such settings. Though data science approaches may be more accessible in our digital future, implementation of TRACERs requires local expertise and commitment to developing data infrastructures. Support for such infrastructure may be more common at large academic medical centers than smaller training sites, potentially perpetuating existing disparities across training programs. Aligning TRACER development with ongoing local and national Patient Safety / Quality Improvement (PSQI) efforts would allow sharing of expertise and infrastructure [37]. For example, some CDS tools and EHR-integrated BPAs may be suitable for development into TRACERs through attention to attribution. Conversely, TRACERs developed in a formative context that show large deviance from guideline-aligned behavior may be good targets for novel CDS tools and BPAs. Linking TRACERs and PSQI efforts may also enable access to federal funding streams to support the necessary work to collect validity evidence for these novel measures. Finally, TRACERs are currently envisioned as an individual-focused assessment, however, one that acknowledges that most patient care is collaborative and team-based [27,53,58]. Future research should continue to explore this tension by considering both attributable actions of individuals as well as how those actions impact the performance of others in the healthcare team.

## Summary

TRACERs are a scalable approach for generating high-density data about trainees that links education to patient care. TRACERs complement existing assessment tools, building on the scalability and automation of the CPM movement to retain focus on individual trainees, while also acknowledging their role within healthcare teams and systems. We believe that TRACERs could form an integral part of assessment systems, providing scalable patient care-focused outcomes to drive CBME forward.
